# Intra thyroid thymic carcinoma: A case report and literature review

**DOI:** 10.1016/j.ijscr.2024.109762

**Published:** 2024-05-17

**Authors:** Han Thi Pham, Hoa Phuong Nguyen, Chu Van Nguyen, Tu Van Dao, Anh Viet Nguyen, Uyen Thi Le

**Affiliations:** a Quansu pathology and cytology department, Vietnam National Cancer Hospital, Campus 1, 43 Quansu Str., Hoankiem Dist., Hanoi, Viet Nam; b Head and Neck Radiotherapy Department, Vietnam National Cancer Hospital, Campus 3, 30 Caubuou Str., Thanhtri Dist., Hanoi, Viet Nam; c High-Quality Treatment Department, Vietnam National Cancer Hospital, Campus 1, 43 Quan Su, Hoan Kiem, Hanoi

**Keywords:** Intrathyroid thymic carcinoma, Thyroid cancers, Case report

## Abstract

**Introduction and importance:**

Intrathyroid thymic carcinoma (ITC) is a malignant epithelial tumor with thymic differentiation within the thyroid gland. Its *frequency is* up to 0.15 % of all malignant thyroid tumors. It is frequently a low-grade tumor. The clinical status is often misleading to other more advanced tumors like cervical lymph node metastasis of nonkeratinizing squamous cell carcinoma, undifferentiated variant, dedifferentiated carcinoma, and medullary carcinoma of the thyroid.

**Case preparation:**

The patient came to us with the diagnosis of cervical lymph node metastasis of undifferentiated carcinoma. This patient was first diagnosed with cervical lymph node metastasis in the previous hospital. After having an ITC diagnosis, the patient was operated on the rennet of thyroid glands and had a low dose of radio-chemotherapy for recurrent prevention purposes. It is the first case of such a disease diagnosed at our hospital and also the first case reported in Vietnam*.*

**Clinical discussion:**

ITC is rare and appears similar to all thymic carcinoma variants. The most popular type is squamous carcinoma. Immunohistochemical stains are typical for thymic origin tumors with CD5, CD117 positive. ITC is often negative for monoclonal PAX8 but positive in this case (MRQ-50 clone, Sigma-Aldrich). This finding is an exciting one that should considered*.*

**Conclusion:**

Reporting the case increases the awareness of the disease, especially among Vietnam Doctors and patients.

## Highlights

• Intrathyroid thymic carcinoma is rare and malignant with thymic differentiation.
• ITC is low-grade but often misleading to other, more advanced tumors.
• The patient primarily had a more aggressive diagnosis, squamous cell carcinoma.
• Treatment for the patient is less aggressive with low-dose chemo-radio therapies.

## 1 Introduction

Intrathyroid thymic carcinoma (ITC) is a pernicious thyroid carcinoma with thymic epithelial differentiation. It is a sporadic carcinoma with an incidence that accounts for up to 0.15 % of all malignant thyroid tumors [1]. The etiology is unknown. It may arise from remnants of the brachial pouch with thymic differentiation or the ectopic thymic in the neck from the embryo stage [1,2]. Though generally to be a low–grade tumor, it is often erroneously diagnosed as a more aggressive tumor-like squamous cell carcinoma, dedifferentiated carcinoma, or cervical metastases disease. The reason is that its morphology is similar to those tumors, and the tumor is uncommon, so not many people are aware of it.

## 2 Case report

The patient was a female, 35 years old, who was admitted to the hospital in January 2024 with the primary diagnosis of cervical lymph node metastasis of undifferentiated carcinoma with a subtotal thyroid operation. Initially, the patient underwent the surgery with the diagnosis of a thyroid tumor suspicious of malignancy.

Return to her history, a thyroid ultrasound of the patient in Dec. 2022 showed a heterogenous hypoechoic solid nodule in the right lower lobe (sized 8x9mm). In addition, a cystic mass was detected in the anterior cervical region, caudal to the thyroid gland, above the superior border of the manubrium sternum. Some lymph nodes were in the right mid and lower jugular. The cytology result of the thyroid nodule was benign. No abnormal family history was detected.

**Fig. 1:**
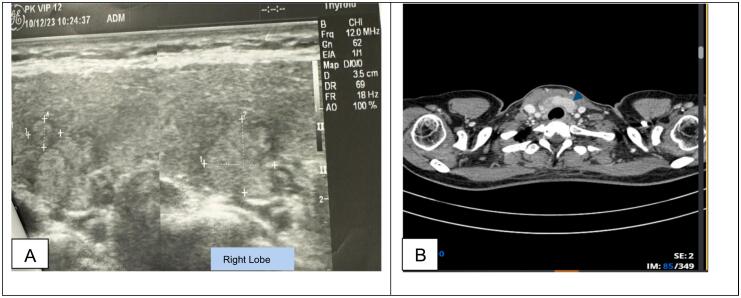
Ultrasound and CECT images of the patent: (A) The ultrasound revealed a large solid mass (38x25mm) in the anterior cervical region, with an ill-defined margin to the right thyroid, classified as a TI-RADS 5 thyroid nodule due to the extension of the lesion with some suspicious cervical lymph nodes, which the largest lymph node was 8x5mm in size. (B). On the non-contrast CT-Scanner images, a heterogenous hyperenhancement solid mass was detected at the same region, above the superior border of the manubrium sternum bone, anterior to the trachea, brachiocephalic vein, caudal to the thyroid, and invaded to the right thyroid lobe, with mild infiltration to the surrounding fat tissue. Some suspicious hyper-enhanced lymph nodes (level VI) were detected; the largest one was 14 × 8 mm in size.

One year later, in Dec. 2023, the ultrasound examination of the same patient showed a large solid mass (38x25mm) in the anterior cervical region, with an ill-defined margin to the right thyroid. The patient was diagnosed with TI-RADS 5 thyroid nodules on ultrasound due to the extension of the lesion with some suspicious cervical lymph nodes, in which the largest lymph node was 8x5mm in size (Fig. 1A).

On the non-contrast CT-Scanner images, a solid mass was detected at the same region, above the superior border of the manubrium sternum bone, anterior to the trachea, brachiocephalic vein, caudal to the thyroid, and invaded the right thyroid lobe. On contrast-enhanced CT, the lesion showed heterogeneous hyperenhancement with mild infiltration to the surrounding fat tissue. Some lymph nodes in the level VI region with the same post-contrast characteristics were detected; the largest was 14 × 8 mm (Fig. 1B). The cytology result of the tumor was carcinoma suspicion.

The patient then underwent the surgery. In the surgery, a tumor measured 30 × 40 mm upper on the sternum, attached to the lower part of the right thyroid lobe. The patient was excited in the right lobe, isthmus, and 1/3 of the lower part of the left lobe. The initial diagnosis was carcinoma metastasized lymph nodes. However, MRI and throat endoscopic detected no tumor.

**Fig. 2:**
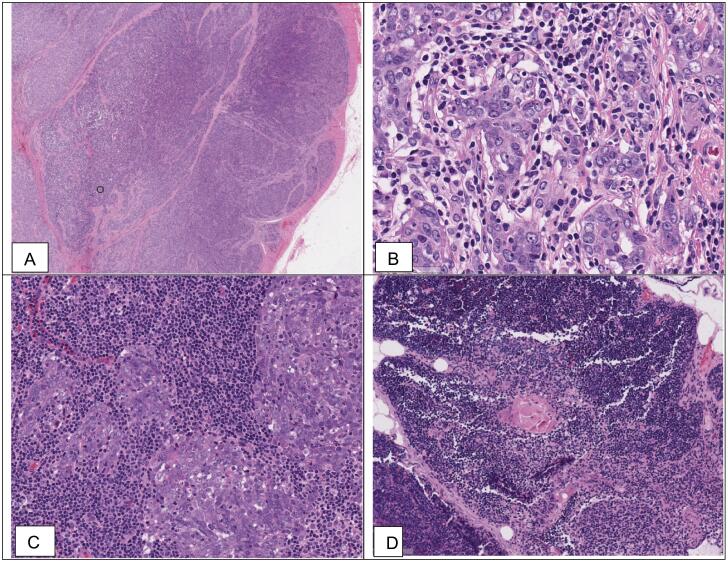
The patient's microscopic features: (A). The tumor was lobulated and separated by thick collagen bundles. The tumor was well-circumscribed and had some satellite nodes (H&E stain, 100×). (B). Oval and round, large tumor cells aggregated in social appearances. They were separated by thick collagen bundles and intermingled with small lymphocytes and plasma cells (H&E stain, 400×). (C). The adjacent lymph node was metastasized by identical tumor cells (H&E stain, 400×). (D) Normal thymic tissues were present adjacent to the thyroid gland (H&E stain, 200×).

Then, the patient came to our hospital for a second opinion. On microscopically, the tumor had a circumscribed border, separated from adjacent surroundings by a thick collagenous bundle pseudo capsule (Fig. 2A). The tumor was composed of large oval to spindle cell tumors. The N/C ratio of the tumor was high with hyperchromasia and scanty cytoplasm. The nuclei were round, coarse chromatin, and prominent nucleoli. Many of the neoplastic cells were aggregated in compound cells. The stromal was dense and collagenous, separating the tumor into lobes. Small lymphocytes and plasmacytoid cells intermingled in the tumor cell individually or patchy (Fig. 2B). These findings suggested a lymphoepithelioma subtype of nonkeratinizing squamous cell carcinoma of the thyroid or dedifferentiated carcinoma. However, the mitotic rate was low and lacked heterogenous nuclear variants and other high-grade nuclear features. The tumor was next to the thyroid gland with Hashimoto's chronic thyroiditis. The identical tumor metastasized one out of two adjacent. Interestingly, some typical thymic foci were present next to the normal thyroid. These findings suggested another discrete diagnosis, intrathyroidal thymic carcinoma.

**Fig. 3:**
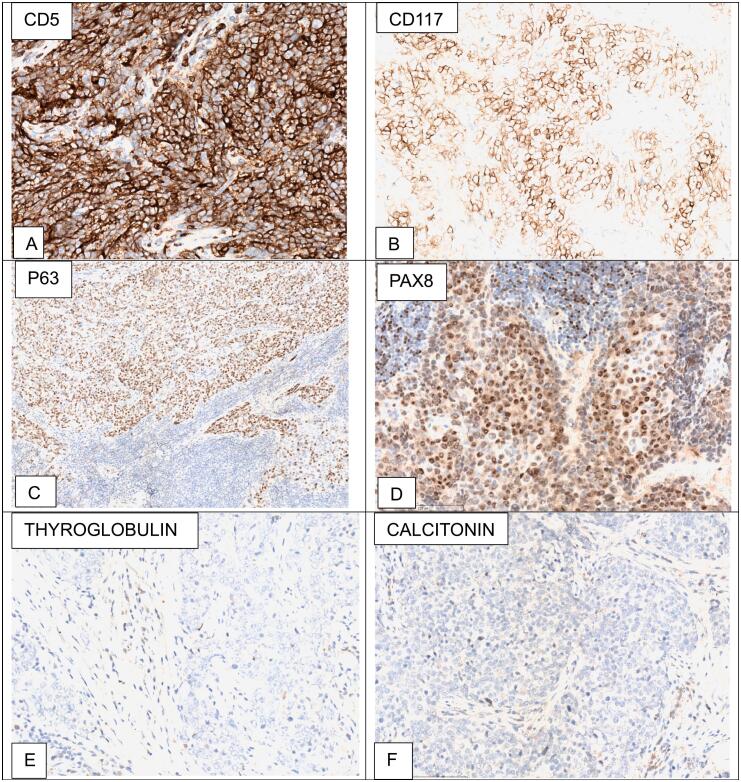
The Immunohistochemical characteristics of the patient: (A) CD5 is robust and diffuse positive (Immunohistochemical stain, 400×). (B) CD117 is moderate and diffuse positive (Immunohistochemical stain, 400×). (C) P63 is strong and diffuse positive (Immunohistochemical stain, 200×). (D) PAX8 is mild and patchy positive (Immunohistochemical stain, 200×). (E), (F) Thyroglobulin and calcitonin are negative (Immunohistochemical stain, 200×).

In immunohistochemical stains, neoplastic cells within tumor islands were strong and diffusely positive for CD5 and Bcl2. p63, CD117 were moderate and heterogeneously (Fig. 3A,B,C). Monoclonal antibody PAX8 (MRQ-50, Sigma-Aldrich) was moderate and patchy positive (Fig. 3D). Thyroglobulin (suggesting follicular thyroid cell tumors) and calcitonin (medullary carcinoma) were negative (Fig. 3E, F). Thyroid function were normal.

Those morphologic and immunohistochemical findings favored intra-thyroid thymic carcinoma, lymphoepithelial subtype.

After having the final diagnosis, the patient underwent total thyroid excision. The leftover of the thyroid had no tumor and had been chronically inflamed. PET/CT has rechecked the patient and has not detected the FDG uptake feature anywhere. MRI and ultrasound have not detected abnormal head, neck, abdomen, and thorax signs. The staging of the disease was pT3N1M0. The patient will then be treated with chemotherapy and radiotherapy simultaneously. The EP recipe used etoposide 120 mg/m2 skin and cisplatin 60 mg/m2 skin in chemotherapy. This treatment was a low-dose recipe for radiotherapy adjuvant and recurrent prevention purposes. The patient also received a low dose of radiotherapy to prevent recurrence ability. The dose of radiotherapy was 54Gy with 1.8 Gy per one time.

## 3 Statement

This case has been reported in line with the SCARE criteria [3].

## 4 Discussion

ITC is a primary malignant epithelial tumor with thymic epithelial differentiation. It is a rare neoplasm, accounting for up to 0,15 % of all malignant thyroid tumors [1]. Pathogenesis is thought to have evolved from ectopic thymic tissue in the thyroid solid cell nests or the remnants of the brachial pouch. These cells remained able to develop into thymic-originated lesions [2]. The tumor often affects middle-aged adults; the median is 52 years old and ranges from 25 to 79 years old. F/M ratio is nearly equal with slight female predominance (1.2:1) [4].

In terms of this case, this is the first case reported in Vietnam. Cancer registration in Vietnam has been established in recent years, but it has not yet been noted [5]. This patient is 35 years old and fit for the etiology of this disease. Due to its rarity, sometimes it is misleading to other more aggressive tumors. This patient was first thought to be lymph node metastasis before admission to our hospital. The reason for their inference is that the tumor is close to the thyroid tissue, and they have numerous lymphocytes around undifferentiated appearance tumor sheets. They referred her to us for further examination. However, PET-CT, CT-scanner, and ENT endoscopy did not detect other abnormalities outside the thyroid region.

Microscopically, the tumor appearance was similar to thymic carcinoma with all their variants, like those in the mediastinum. The most common phenotype is thymic squamous carcinoma [2,4]. In this tumor, neoplastic cells comprise squamoid and focally spindle and compounded cells with lightly eosinophilic cytoplasm. Nuclei were round to oval with bland to vesicular nuclear chromatin. Sometimes, single-cell keratinization was present. The stroma was sclerotic with thick collagen bands. Haphazardly, small lymphocytes and plasmocytes were present in the tumor. The tumor has a clear boundary with the collagenous capsule.

Interestingly, some typical thymic tissue areas were present next to the tumor and hosted thyroid glands. This finding confirmed the theory that the tumor developed from the ectopic thymic tissue in the thyroid [2,4]. The tumor was next to the thyroid tissue with chronic lymphoid thyroiditis. The identical tumor metastasized in two out of two adjacent lymph nodes. Stimulating spindle and oval tumor cells effaced most of the lymph nodes.

Immunohistochemical stains revealed with CD5 and CD117 were positive. It is partially positive with PAX8, P63, and TTF1. It was negative with thyroglobulin and calcitonin. Suzuki [6] suggests using the monoclonal antibody PAX8 (paired-box gene 8, clone EPR13510, Abcam) to distinguish intrathyroidal thymic carcinoma from follicular cell-derived thyroid carcinomas, including thyroid squamous cell carcinoma and anaplastic carcinoma. Paired-box gene 8 (PAX8) is a transcription factor essential for embryonic thyroid development [7]. Thyroid follicular cell origin carcinomas, including squamous and anaplastic carcinoma, are reported to be immunoreactive for PAX8. In Wang's study [8], all ten cases of ITCs were nuclear positive with PAX8 (polyclonal, ProteinTech Group). Toriyama [9] suggested that polyclonal PAX8 antibody immunoreactions are likely due to the cross-reactivity between PAX5 and PAX6. However, Suzuki [6] and Baloch [4] pointed out that monoclonal PAX8 is negative in intrathyroid thymic carcinomas. In Suzuki's study, four out of ten cases of ITCs were positive with polyclonal PAX8 (Polyclonal clone, Proteintech), while none of them was positive with monoclonal PAX8 (EPR13510, Abcam). However, our patient was partially nuclear reactive with a monoclonal PAX8 (MRQ-50, Sigma-Aldrich). This is an exciting finding that should be noticed.

Operation is the best choice for ITC because local recurrence and lymph node metastasis are popular. Adjuvant radiotherapy is reported to be favored because of radiosensitivity. A low dose of chemo-radio therapy after surgery is recommended for reducing recurrence [10]. Some chemotherapy regimes have been applied to ITC, for example, cisplatin, liposomal doxorubicin, epirubicin, docetaxel, gemcitabin, and irinotecan [11]. This patient was treated with etoposide and cisplatin simultaneously with radiotherapy. The two chemo agents have been proven to be effective in chemoradiotherapy [12]. However, there is no definitive treatment strategy for this disease now, so ITC patients should be planned personally according to disease stage and patient's condition [8,12].

## 5 Conclusion

In conclusion, ITC is a rare carcinoma with complex histomorphology and immunohistochemical characteristics. The diagnosis of ITC is challenging, especially in Vietnam, where other carcinomas, especially squamous cell carcinoma, are far more outstanding than ITC in the head and neck, as well as it has not been yet informed before. The patient is the first case reported in Vietnam. The histopathology of the tumor in this patient is similar to other ITC. The immunohistochemical staining is consistent with CD5, CD117, and p40. Monoclonal PAX8 antibody (MRQ-50, Sigmal-Aldrich) was partially nuclear immunoreactive in this patient and should be considered in practice. These findings expanded our knowledge of ITC. The treatment for the disease differs from case to case and should depend on the stage of the disease and the patient basis.

## Ethics approval

Ethical approval is not required for this study under local guidelines. However, We have had the patient's consent to publish details of the patient's medical case and any accompanying images. She understood that her name and initials would not be published.

## Funding

The authors received no financial support for this article's research, authorship, and publication.

## Author contribution

All authors contributed equally to the study. Dr. Han Thi Pham and Chu Van Nguyen have diagnosed the case, designed the concept, and written the paper. Ms. Hoa Phuong Nguyen and Dr. Uyen Thi Le collected data. Dr. Tu Van Dao and Anh Viet Nguyen collected and analyzed the case.

## Guarantor

Dr. Han Thi Pham is the guarantor for this study. Please do not hesitate to contact Dr. Han Thi Pham for any questions via email: phamthihan.bvk@gmail.com or mobile phone: (+84) 988091635.

## Research registration number

This report is not the “First in Man” study.

## Consent

Written informed consent was obtained from the patient for publication of this case report and accompanying images. A copy of the written consent is available for review by the Editor-in-Chief of this journal on request. The authors have omitted the patient's name and initials, as well as our hospital information. We declare that we will protect the patient's anonymity.

## Declaration of competing interest

The authors declare no potential conflicts of interest concerning this article's research, authorship, and publication.
